# Communication in Primary Healthcare: A State-of-the-Art Literature Review of Conversation-Analytic Research

**DOI:** 10.1080/08351813.2024.2305038

**Published:** 2024-04-03

**Authors:** Rebecca K. Barnes, Catherine J. Woods

**Affiliations:** a Nuffield Department of Primary Care Health Sciences, University of Oxford, Radcliffe Observatory Quarter, U.K.; b School of Primary Care, Population Sciences and Medical Education, University of Southampton, Aldermoor Health Centre, U.K.

## Abstract

We report the first state-of-the-art review of conversation-analytic (CA) research on communication in primary healthcare. We conducted a systematic search across multiple bibliographic databases and specialist sources and employed backward and forward citation tracking. We included 177 empirical studies spanning four decades of research and 16 different countries/health systems, with data in 17 languages. The majority of studies originated in United States and United Kingdom and focused on medical visits between physicians and adult patients. We generated three broad research themes in order to synthesize the study findings: managing agendas, managing participation, and managing authority. We characterize the state-of-the-art for each theme, illustrating the progression of the work and making comparisons across different languages and health systems, where possible. We consider practical applications of the findings, reflect on the state of current knowledge, and suggest some directions for future research. Data reported are in multiple languages.

## Overview and background

Primary healthcare (PHC) is a key element of health systems in many countries, distinct from other aspects of health services delivery systems, such as specialty outpatient care (see Ekberg et al., 2024) or emergency medical care (see Riou, 2024). PHC is widely recognized as the “front door” into health care systems, providing ambulatory or first-contact personal healthcare services, and often performing a gate-keeping role. It is rooted ostensibly in a commitment to social justice, equity, and participation:

PHC is a whole-of-society approach to health that aims at ensuring the highest possible level of health and well-being and their equitable distribution by focusing on people’s needs and as early as possible along the continuum from health promotion and disease prevention to treatment, rehabilitation and palliative care, and as close as feasible to people’s everyday environment. (WHO & UNICEF, 2018, p. xii)

In the United Kingdom, for example, PHC involves treatment for acute minor illnesses, and the management of long-term conditions such as diabetes and heart disease, as well as the prevention of potential future ill-health through health promotion, immunization, and screening programmes.

Comprehensive generalist physicians (i.e., general practitioners or family physicians) and nurses play a central role as trained specialists in PHC. In some healthcare systems, other medical specialists (e.g., internists specializing in adult internal medicine and pediatricians) play a key role. Primary care teams may also include pharmacists, paramedics, physiotherapists, occupational therapists, midwives, physician assistants/associates, community health workers, and dentists.

In this review, we focus on conversation-analytic (CA) studies of the provision of treatment for acute minor illnesses and the management of long-term conditions. Our rationale is purely pragmatic: A review of all CA studies meeting the wide remit of PHC as described above would be difficult to contain. Moreover, such heterogeneity would reduce the possibility of identifying systematic and differential practices of action, and of making useful comparisons across countries and languages (see inclusion and exclusion criteria). Within these parameters, the aim of our review was to perform a comprehensive search of the historical and current literature. In what follows we describe our methods and present a narrative synthesis of the findings. We then consider practical applications, reflect on our approach to the review and the strengths and weaknesses of the field to-date, and suggest some priorities for future research.

## Methods

Our first step was a scoping search that included existing or ongoing reviews of CA in PHC. After reviewing the coverage, relevance, and accuracy of the search findings with an information specialist, we decided on the following search terms: “conversation analysis,” “medicine,” and “primary health care.” We ran this search across the following bibliographic databases: Cinahl, Embase, Medline, PsychINFO, SSCI, and Scopus. No date restrictions were employed. To ensure the inclusion of all relevant material (e.g., book chapters), we consulted the EM/CA wiki^1^^1^See https://emcawiki.net/EMCA_bibliography_database. bibliography of medical CA studies and searched our personal collections, online contents of popular journals in the field, and checked the publication pages of key scholars. The final search date was March 11, 2022.

All results were exported into Rayyan,^2^^2^See Ouzzani et al. (2016). a collaborative review software package, and any duplicates removed. Next, titles and abstracts were screened independently by both authors against the following inclusion and exclusion criteria.

Inclusion criteria

1. CA studies of communication in PHC (including face-to-face and telephone consultations, new and follow-up acute care appointments, routine chronic care appointments, annual checkups, new patient appointments, and urgent primary care);
2. published journal articles (including pre-prints where available), book chapters, and official reports;
3. all countries of origin; and
4. all languages where final publication is in English.

Exclusion criteria

1. studies of preventative care (including vaccinations), physical rehabilitation, or palliative care;
2. studies of wider PHC (e.g., maternity services, community mental health services, community pharmacy, physiotherapy services, dentistry, audiology, and optometry);
3. studies of manipulated PHC practice (i.e., simulated data or data from primary care trials);
4. studies of interactions with trainees (e.g., medical students) or support staff (e.g., interpreters, receptionists);
5. studies comparing PHC data with data from other healthcare settings;
6. monographs, literature reviews, commentaries, editorials, and conference abstracts; and
7. masters’ dissertations or Ph.D. theses.

Any conflicts were highlighted in Rayyan and resolved during regular meetings between the two authors. Full texts were retrieved for the remaining studies. When articles or book chapters were unavailable online, we sought copies from corresponding authors or requested interlibrary loans. All full texts were listed alphabetically and then split equally between the authors for closer screening against our criteria. All excluded texts were labeled in Rayyan with reasons for exclusion and any uncertainties flagged as “maybe” for subsequent discussion (e.g., if we were unsure if the context counted as PHC). Once we had agreed on our final included studies, we used Google Scholar to conduct forward and backward citation tracking to identify any further studies for screening. On completion, 177 studies were included for analysis.

We developed an Excel table to organize data extraction. For the purposes of this review, we did not assess the quality of our included studies. Where evident, studies using the same dataset were highlighted, and missing information was sought from other sources. We tabulated studies chronologically to track development of the field over time. The study findings were imported into NVivo. Codes were created, applied, and refined, and from these we generated three themes. Our decision -making here was informed by (1) the desire to bring together systematic and differential practices of action identified and (2) a wish to contextualize the findings, where possible, with topics in the wider PHC literature and/or professional guidance.

## Current state-of-the-art

A total of 177 CA studies of PHC published between 1981 and 2022 were included. Of these, 137 were peer-reviewed research articles (90 from medical or health-related journals), and 40 were chapters in edited books. The studies originated from 16 countries, dominated by the United States (*n* = 69) and the United Kingdom (*n* = 52), with data in 17 different languages (see Table 1).

**Table 1.** Included studies.

	Author/s	Year	Type	Country	HCPs	No.	Patients	No.	Theme 1 - Managing agendas 2 - Managing participation 3 - Managing authority	Data language
1	Heath	1981	Book chapter	UK	Physicians	*	*	4000^i^	Managing agendas	British English
2	Meehan	1981	Book chapter	US	Physicians	*	*	*	Managing authority	American English
3	Heath	1982	Article	UK	Physicians	*	*	*	Managing participation	British English
4	Paget	1983	Book chapter	US	Physicians	1	Adults	1	Managing authority	American English
5	West	1983	Book chapter	US	Physicians	18	Mixed	20	Managing authority	American English
6	Heath	1984a	Article	UK	Physicians	*	*	*	Managing participation	British English
7	Heath	1984b	Book chapter	UK	Physicians	*	*	*	Managing participation	British English
8	West	1984	Article	US	Physicians	18	Mixed	20	Managing authority	American English
9	Heath	1985	Article	UK	Physicians	*	*	500^i^	Managing participation	British English
10	Houtkoop-Steenstra	1986	Book chapter	NL	Physicians	1	Adults	1	Managing participation	Dutch
11	Freeman	1987	Article	US	Physicians	*	Adults	200^i^	Managing authority	American English
12	Heath	1989	Article	UK	Physicians	*	*	1000^i^	Managing participation	British English
13	Frankel	1990	Book chapter	US	Physicians	*	Adults	10	Managing authority	American English
14	West	1990	Article	US	Physicians	18	Mixed	20	Managing authority	American English
15	ten Have	1991	Book chapter	NL	Physicians	*	*	*	Managing authority	Dutch
16	Heath	1992	Book chapter	UK	Physicians	*	*	*	Managing authority	British English
17	Greatbatch et al.	1993	Article	UK	Physicians	4	*	200^i^	Managing participation	British English
18	Mulholland	1994	Article	AU	Physicians	*	*	*	Managing authority	Australian English
19	Greatbatch et al.	1995	Article	UK	Physicians	4	*	250	Managing participation	British English
20	ten Have	1995	Book chapter	NL	Physicians	*	*	4	Managing authority	British English & Dutch
21	Gill	1998	Article	US	Physicians	4	Adults	15	Managing agendas	American English
22	Peräkylä	1998	Article	FI	Physicians	14	*	100^i^	Managing authority	Finnish
23	Robinson	1998	Article	US	Physicians	*	Mixed	86	Managing participation	British English
24	Heritage & Stivers	1999	Article	US	Physicians	19	Mixed	335	Managing authority	American English
25	Joosten et al.	1999	Article	NL	Physicians	8	Adults	24	Managing participation	Dutch
26	Gill et al.	2001	Article	US	Physicians	1	Adult	1	Managing agendas	American English
27	Haakana	2001	Article	FI	Physicians	*	*	60	Managing participation	Finnish
28	Jones	2001	Article	US	Physicians	11	*	25	Managing participation	American English
29	Robinson	2001a	Article	US	Physicians	7	Adult	48	Managing agendas	American English
30	Robinson	2001b	Article	US	Physicians	1	Adult	1	Managing authority	American English
31	Robinson & Stivers	2001	Article	US	Physicians	8	*	40	Managing participation	American English
32	Ruusuvuori	2001	Article	FI	Physicians	10	*	100^i^	Managing participation	Finnish
33	Stivers	2001	Article	US	Physicians	13	Children	100	Managing participation	American English
34	Stivers & Heritage	2001	Article	US	Physicians	1	Adult	1	Managing authority	American English
35	Heath	2002	Article	UK	Physicians	*	*	2000^i^	Managing authority	British English
36	Manning & Ray	2002	Article	US	Physicians	*	*	22	Managing agendas	American English
37	Peräkylä	2002	Article	FI	Physicians	14	*	100^i^	Managing authority	Finnish
38	Stivers	2002a	Article	US	Physicians	14	Children	360	Managing agendas	American English
39	Stivers	2002b	Article	US	Physicians	14	Children	360	Managing agendas	American English
40	Gafaranga & Britten	2003	Article	UK	Physicians	20	*	62	Managing agendas	British English
41	Mangione-Smith et al.	2003	Article	US	Physicians	10	Children	306	Managing authority	American English
42	Maynard & Frankel	2003	Book chapter	US	Physicians	1	Adults	1	Managing authority	American English
43	Modaff	2003	Book chapter	US	Physicians	*	Mixed	*	Managing participation	American English
44	Pilnick & Coleman	2003	Article	UK	Physicians	29	Adult	47	Managing authority	British English
45	Robinson	2003	Article	US	Physicians	9	Adult	69	Managing participation	American English
46	Stivers et al.	2003	Article	US	Physicians	8	Children	295	Managing agendas	American English
47	Britten et al.	2004	Article	UK	Physicians	20	Mixed	35	Managing participation	British English
48	Campion & Langdon	2004	Article	UK	Physicians	9	*	237	Managing agendas	British English
49	Gafaranga & Britten	2004	Article	UK	Physicians	20	*	62	Managing participation	British English
50	Lutfey	2004	Article	US	Nurses & Physicians	*	*	26	Managing authority	American English
51	Pomerantz & Rintel	2004	Article	US	Physicians	*	*	33	Managing authority	American English
52	Collins	2005	Article	UK	Nurses & Physicians	11	*	23	Managing authority	British English
53	Gafaranga & Britten	2005	Book chapter	UK	Physicians	*	*	62^i^	Managing agendas	British English
54	Gill	2005	Article	US	Physicians	1	Adult	1	Managing agendas	American English
55	Kitzinger	2005	Article	UK	Physicians	1	*	50	Managing participation	British English
56	Leppänen	2005	Book chapter	SE	Nurses	13	*	209	Managing agendas	Swedish
57	Robinson & Heritage	2005	Article	US	Physicians	77	*	302	Managing agendas	American English
58	Stivers	2005a	Article	US	Physicians	*	Children	309	Managing authority	American English
59	Stivers	2005b	Article	US	Physicians	14	Children	360	Managing authority	American English
60	Wynn	2005	Article	NO	Physicians	3	Adults	77	Managing participation	British English
61	Boyd & Heritage	2006	Book chapter	US	Physicians	1	Adults	1	Managing agendas	American English
62	Drew	2006	Book chapter	UK	Physicians	1	*	60	Managing agendas	British English
63	Gill & Maynard	2006	Book chapter	US	Physicians	5	Adults	15	Managing agendas	American English
64	Greatbatch	2006	Book chapter	UK	Physicians	4	*	80	Managing participation	British English
65	Halkowski	2006	Book chapter	US	Physicians	*	*	25	Managing agendas	American English
66	Heath	2006	Book chapter	UK	Physicians	*	*	*	Managing participation	British English
67	Heritage & Robinson	2006a	Article	US	Physicians	77	Adults	302	Managing agendas	American English
68	Heritage & Robinson	2006b	Book chapter	US	Physicians	*	*	300^i^	Managing agendas	American English
69	Mangione-Smith et al.	2006	Article	US	Physicians	38	Children	522	Managing authority	American English
70	Maynard & Frankel	2006	Book chapter	US	Physicians	*	*	*	Managing authority	American English
71	Peräkylä	2006	Book chapter	FI	Physicians	14	*	71	Managing authority	Finnish
72	Pillet-Shore	2006	Article	US	Nurses	*	Adults	14	Managing authority	American English
73	Pilnick & Coleman	2006	Article	UK	Physicians	29	Adults	47	Managing authority	British English
74	Rhodes et al.	2006	Article	UK	Nurses	1	Adults	1	Managing participation	British English
75	Robinson & Heritage	2006	Article	US	Physicians	28	Adults	142	Managing agendas	American English
76	Robinson	2006	Book chapter	US	Physicians	*	Adults	182	Managing agendas	American English
77	Sorjonen et al.	2006	Book chapter	FI	Physicians	*	Adults	25	Managing authority	Finnish
78	Stivers	2006	Book chapter	US	Physicians	*	*	*	Managing authority	American English
79	West	2006	Book chapter	US	Physicians	*	Mixed	62	Managing agendas	American English
80	Cahill & Papageorgiou	2007	Article	UK	Physicians	16	Children	31	Managing participation	British English
81	Gafaranga & Britten	2007	Book chapter	UK	Physicians	*	*	*	Managing participation	British English
82	Pomerantz et al.	2007	Book chapter	US	Physicians	*	Adults	3	Managing agendas	American English
83	Stivers & Majid	2007	Article	US	Physicians	*	Children	322	Managing participation	American English
84	Dew et al.	2008	Article	NZ	Physicians	*	Adults	9	Managing authority	New Zealand English
85	Rhodes et al.	2008	Article	UK	Nurses & Physicians	13	Adults	26	Managing participation	British English
86	Ariss	2009	Article	UK	Physicians	4	Adults	13	Managing agendas	British English
87	Frers	2009	Article	DE	Physicians	*	*	*	Managing participation	American English
88	Cahill	2010	Book chapter	UK	Physicians	16	Children	31	Managing participation	British English
89	Gill et al.	2010	Article	US	Physicians	*	*	50	Managing agendas	American English
90	Heritage et al.	2010	Article	US	Physicians	38	Children	522	Managing authority	American English
91	Hewitt et al.	2010	Article	UK	Physicians	18	Adults	65	Managing participation	British English
92	Ijäs-Kallio et al.	2010a	Article	FI	Physicians	11	Mixed	86	Managing authority	Finnish
93	Ijäs-Kallio et al.	2010b	Article	FI	Physicians	*	Mixed	10	Managing agendas	Finnish
94	Leppänen	2010	Book chapter	SE	Nurses	13	Mixed	276	Managing participation	Swedish
95	Newman et al.	2010	Article	UK	Physicians	4	Mixed	52	Managing participation	British English
96	Pilnick & Coleman	2010	Article	UK	Physicians	29	Adults	47	Managing authority	British English
97	Cohen et al.	2011	Article	US	Physicians	*	Adults	541	Managing authority	American English
98	Ijäs-Kallio et al.	2011	Article	FI	Physicians	*	Mixed	53	Managing participation	Finnish
99	Koenig	2011	Article	US	Physicians	*	Adults	100	Managing authority	American English
100	Beck Nielsen	2011	Article	DK	Physicians	*	*	9	Managing agendas	Danish
101	Park	2011	Article	KR	Physicians	*	*	*	Managing participation	Korean
102	Denvir	2012	Article	US	Physicians	6	Adults	24	Managing authority	American English
103	Dillon	2012	Article	US	Physicians	15	Adults	96	Managing authority	American English
104	Beck Nielsen	2012	Article	DK	Physicians	4	*	18	Managing agendas	Danish
105	Stivers	2012	Article	US	Physicians	*	Children	322	Managing participation	American English
106	Bergen & Stivers	2013	Article	US	Physicians	*	Adults	57	Managing agendas	American English
107	Dowell et al.	2013	Article	NZ	Physicians	10	Adults	28	Managing participation	New Zealand English
108	Halkowski	2013	Book chapter	US	Physicians	*	*	100	Managing authority	American English
109	Miller	2013	Article	UK	Physicians	*	*	*	Managing participation	British English
110	Park	2013	Article	KR	Physicians	7	*	60	Managing agendas	Korean
111	Chatwin et al.	2014	Article	UK	Nurses & Physicians	*	*	26	Managing agendas	British English
112	Beck Nielsen	2014	Book chapter	DK	Physicians	4	*	52	Managing participation	Danish
113	Wingard et al.	2014	Book chapter	US	Physicians	*	*	55	Managing authority	American English
114	Guassora et al.	2015	Article	DK	Physicians	6	*	15	Managing authority	Danish
115	Guzmán	2015	Article	CL	Healers & Physicians	6	Children	58	Managing authority	Mapudungun & Spanish
116	Mangione-Smith et al.	2015	Article	US	Physicians	28	Children	1194	Managing authority	American English
117	Tarber	2015	Article	DK	Physicians	*	Mixed	3	Managing agendas	Danish
118	Tarber & Frostholm	2015	Article	DK	Physicians	*	Adults	4	Managing agendas	Danish
119	Wheat et al.	2015	Article	UK	Physicians	*	Adults	26	Managing authority	British English
120	Barton et al.	2016	Article	NZ	Allied HCPs, Nurses & Physicians	*	*	3	Managing authority	New Zealand English
121	Beck Nielsen	2016	Article	DK	Physicians	4	*	52	Managing participation	Danish
122	Robinson et al.	2016	Article	US	Physicians	85	Adults	407	Managing agendas	American English
123	Tarber et al.	2016	Article	DK	Physicians	1	Adults	1	Managing authority	Danish
124	Vickers et al.	2016	Article	US	Nurses	3	Adults	50	Managing participation	American English & Spanish
125	Lindell	2017	Article	DK	Physicians	10	Mixed	31	Managing participation	Danish
126	Park	2017	Article	KR	Physicians	*	*	24	Managing agendas	Korean
127	Wu	2017	Article	CN	Physicians	*	*	100^i^	Managing authority	Mandarin
128	Barnes	2018	Article	UK	Physicians	*	Adults	57	Managing participation	British English
129	Bergen et al.	2018	Article	US	Physicians	93	Adults	*	Managing authority	American & British English
130	Heath	2018	Book chapter	UK	Physicians	*	*	*	Managing participation	British English
131	Lenzen et al.	2018	Article	NL	Nurses	3	Adults	5	Managing participation	Dutch
132	McArthur	2018	Article	US	Physicians	*	*	171	Managing participation	American English
133	Beck Nielsen	2018a	Article	DK	Physicians	*	Adults	18	Managing participation	Danish
134	Beck Nielsen	2018b	Article	DK	Physicians	*	Adults	11	Managing authority	Danish
135	Beck Nielsen	2018c	Article	DK	Physicians	4	Adults	52	Managing participation	Danish
136	Nguyen & Austin	2018a	Article	AU	Physicians	15	Adults	66	Managing authority	Vietnamese
137	Nguyen & Austin	2018b	Article	AU	Physicians	12	Adults	31	Managing agendas	Vietnamese
138	Stivers et al.	2018	Article	US	Physicians	*	*	*	Managing authority	American & British English
139	Abu El-Rob	2019	Article	UK	Physicians	*	*	*	Managing participation	Arabic
140	Antaki & Chinn	2019	Article	UK	Nurses, Physicians, Physician Associate	*	Adults	25	Managing participation	British English
141	Cabral et al.	2019	Article	UK	Nurses & Physicians, Physician’s Assistant	*	Children	56	Managing agendas	British English
142	Cheng	2019	Article	HK	Nurses	5	Adults	61	Managing participation	Cantonese & Mandarin Chinese
143	Chinn	2019	Article	UK	Nurses, Physicians, Physician Associate	18	Adults	41	Managing participation	British English
144	Heritage & McArthur	2019	Journal article	US	Physicians	*	*	201	Managing authority	American English
145	Lindell et al.	2019	Book chapter	DK	Physicians	*	Mixed	43	Managing agendas	Danish
146	McArthur	2019	Article	US	Physicians	*	Adults	255	Managing authority	American English
147	Beck Nielsen	2019a	Article	DK	Physicians	4	Adults	52	Managing participation	Danish
148	Beck Nielsen	2019b	Book chapter	DK	Physicians	1	Adults	1	Managing agendas	Danish
149	Stevenson et al.	2019	Article	UK	Physicians	5	Mixed	18	Managing participation	British English
150	Tate	2019	Article	US	Physicians	*	Adults	14	Managing agendas	American English
151	Wang	2019	Book chapter	CN	Physicians	9	Children	187	Managing participation	Mandarin Chinese
152	Abu El-Rob	2020	Article	UK	Physicians	8	Mixed	20	Managing participation	Arabic
153	Bergen	2020	Article	US	Physicians	*	Adults	*	Managing authority	American English
154	Ford et al.	2020	Article	UK	Physicians	*	Adults	2	Managing participation	British English
155	Kushida et al.	2020	Article	JP	Physicians	*	*	29	Managing agendas	Japanese
156	La & Weatherall	2020	Book chapter	NZ	Physicians	*	*	24	Managing participation	New Zealand English
157	Li	2020	Article	CN	Physicians	*	*	8	Managing authority	Mandarin Chinese
158	Montenegro & Dori-Hacohen	2020	Article	US	Physicians	9	Adults	10	Managing authority	American English
159	Wang	2020	Article	CN	Physicians	*	Children	187	Managing agendas	Mandarin Chinese
160	Arreskov et al.	2021	Article	DK	Physicians	13	Adults	14	Managing agendas	Danish
161	Barnes & van der Scheer	2021	Book chapter	UK	Physicians	*	Adults	24	Managing authority	British English
162	Chinn & Ruddall	2021	Article	UK	Nurses, Physicians, Physician Associate	*	Adults	24	Managing participation	British English
163	Connabeer	2021	Article	UK	Physicians	22	Adults	86	Managing authority	British English
164	Erkelens et al.	2021	Article	NL	Nurses	*	*	68	Managing participation	Dutch
165	Ford et al.	2021	Article	UK	Physicians	*	Adults	18	Managing participation	British English
166	McCabe	2021	Article	UK	Physicians	13	Adults	23	Managing authority	British English
167	Ostermann	2021	Article	BR	Physicians	3	Adults	103	Managing authority	Brazilian Portuguese
168	Stevenson et al.	2021a	Article	UK	Physicians	*	*	19	Managing authority	British English
169	Stevenson et al.	2021b	Article	UK	Physicians	*	Mixed	28	Managing authority	British English
170	Stivers & Timmermans	2021	Article	US	Physicians	*	Adults	68	Managing agendas	American English
171	Stortenbeker et al.	2021	Article	NL	Physicians	10	Adults	14	Managing participation	Dutch
172	Wang & Liu	2021	Article	CN	Physicians	*	Children	183	Managing authority	Mandarin Chinese
173	Weatherall et al.	2021	Article	NZ	Physicians	6	*	9	Managing participation	New Zealand English
174	Chinn	2022	Article	UK	Nurses & Physicians	9	Adults	24	Managing participation	British English
175	Dooley & Barnes	2022	Article	UK	Paramedic & Physicians	4	Adults	17	Managing agendas	British English
176	Tietbohl	2022	Article	US	Physicians	4	Adults	52	Managing participation	American English
177	Tietbohl & Bergen	2022	Article	US	Physicians	14	Adults	90	Managing participation	American English

*Missing data or unclear; iNumber of recorded consultations.

Country of origin abbreviations: AU – Australia; BR – Brazil; CL – Chile; CN – China; DE – Germany; DK – Denmark; FI – Finland; HK – Hong Kong; JP – Japan; KR – Korea; NL – Netherlands; NO – Norway; NZ – New Zealand; SE – Sweden; UK – United Kingdom; US – United States.

The overarching and interrelated broad themes generated were managing agendas, managing participation, and managing authority. Below, we describe each theme, provide illustrative examples, and highlight both similar and, where appropriate, contrasting findings across countries and languages. We also attend to chronology, to allow insight into trends, developments, and trajectories in the field over time.

## Managing agendas

The acute primary care consultation is structurally organized to address a singular “chief” problem or concern; however, patients often present to PHC with multiple agenda items (Leydon et al., 2018). Managing or negotiating agendas is a perennial problem for physicians and patients (Manning & Ray, 2002). If done successfully, it can avoid the potential for “unvoiced” agenda items and maximize efficiency (Barry et al., 2000). In the PHC literature and professional guidance, agenda setting (i.e., soliciting the patient’s agenda for the consultation to reconcile with the physician’s own agenda; see Levenstein et al., 1986), has been championed as a strategy for addressing this problem. It provides an organizing framework for physicians to prioritize which issues can be dealt with, and which should be deferred to another visit or referred elsewhere.

### Physicians’ agenda-setting practices

Examining the practices whereby physicians manage agenda setting has been and continues to be a key research theme in CA studies of PHC. One such practice is to elicit the patient’s chief concern at the outset of the consultation. Indeed, this was the focus of the earliest CA study included in our review (Heath, 1981). Drawing on British data collected throughout the 1970s, Heath characterized the different formats of physicians’ “first topic initiators” or opening questions that solicit the patient’s reason for the visit (Heath, 1981). Distinguishing between “Type A” first topic initiators found in new appointments (e.g., “How can I help?”), and “Type B,” found in return appointments (e.g., “How are you doing?”), Heath demonstrated how physicians’ opening questions were designed for the recipient, providing for the generation of an “appropriate and correct” first topic or “disclosure sequence” (Heath, 1981, p. 78) for medical assessment. Heath’s findings have since been replicated in British data collected in the 1990s (Gafaranga & Britten, 2003); in U.S. data (Robinson, 2006); and, more recently, in Korean (Park, 2017) and Vietnamese data (Nguyen & Austin, 2018).

Building on these findings, several studies have considered the implications of different kinds of Type A opening question formats. For example, in a mixed methods study of physicians’ opening questions in U.S. data, Heritage and Robinson (2006a) found that “general enquiry” questions (e.g., “What can I do for you today?”) were statistically associated with longer patient problem presentations and more positive post-visit evaluations of physicians’ communication (Robinson & Heritage, 2006) than “confirmatory” questions (e.g., “Sore throat, huh?”). Contrastingly, in the Korean data, Park (2017) found physicians most frequently employed, “*Eti-ka pwulphenhasyese osyesseyo?*”—a format that translates as, “Where does it hurt that (made you) come in?” These “location” questions were associated with briefer problem presentations and a faster transition into history taking, possibly reflecting a “culturally specific goal-oriented progressivity” (Park, 2017, p. 14).

Studies of the normative organization of physicians’ opening questions have also provided a framework for understanding “problematic” communication during nonstandard consultation openings. For example, in British data from out-of-hours home visits to patients living with dementia, Dooley and Barnes (2022) observed that physicians opened the consultations by stating their own reason for the visit, using information from case record entries made during the caregiver’s initial call. In Fragment (1), at Line 1 the physician informs the patient of the reason for the visit.

**Fragment (1) From Dooley and Barnes (2022, p. 3).**

**Figure:**
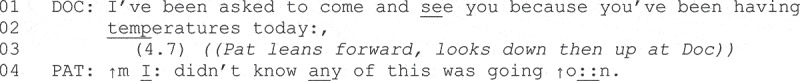

Misalignment was evident in all the home visit openings in the study, as shown in Line 4, where the patient denies the existence of current symptoms and/or their involvement in the decision to seek help.

In professional guidance, upfront agenda setting (i.e., pursuit of further concerns immediately after patients have presented their chief concern) is often recommended in a bid to increase efficiency (Brock et al., 2011). In a mixed methods analysis of three U.S. datasets collected between 2000 and 2005, Robinson et al. (2016) examined the nature, positioning, and effectiveness of different physician questioning strategies designed to elicit additional patient concerns. They found that “concern-seeking” questions asked early in the visit were effective in generating additional agenda items, but were rarely employed (see Heritage et al., 2007).

The closing phase is another location where physicians might elicit further agenda items by initiating a “final-concern sequence.” However, Robinson (2001a) found that the design of physicians’ final-concern questions (e.g., “Any other concerns?”) in U.S. data tended to bias answers toward “no” responses, and that the questions were often delivered during other activities (e.g., while entering information into the patient’s health record), without gazing at the patient, or even while standing up in anticipation of the patient’s exit (Robinson 2001a).

### Patients’ agenda-setting practices

#### Topic initiation

Our included studies also offered distinctive insights into how patients negotiate their own agendas. For example, Heath (1981) noted that patients would sometimes initiate second and occasionally third topics themselves. Campion and Langdon (2004) confirmed this in British data collected in the late 1980s. They also observed that while presenting their reason for the visit, some patients used “pre-announcements” (e.g., “Well, it’s three things really”) or listing practices (e.g., “First of all …”) to give advance warning of candidate second or third topics that their physicians might either accept, postpone, or ignore. More commonly, patients would indicate second or third topics later in the visit via “opportunistic announcements,” following observable breaks in the physician’s attention or during the closing phase of the visit. Campion and Langdon (2004) noted that the success of such later attempts were also subject to their acknowledgment and consideration by physicians.

Similarly, in Danish data, Beck Nielsen (2012) found that patients would attempt to initiate new topics later in the visit, in response to physicians’ closing-implicative moves such as “arrangement-making sequences,” as shown in Fragment (2):

**Fragment (2) From Beck Nielsen (2012, p. 557).**

**Figure:**
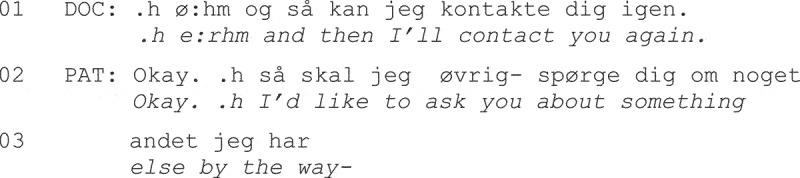

Beck Nielsen (2012) found that all additional concerns initiated by patients were addressed by physicians. Contrastingly, in Korean data, Park (2013) found that physicians would push toward closure rather than engaging with patients’ attempts to topicalize additional concerns, on the rare occasion patients did so.

#### Patients’ ideas

Patients’ agenda-setting practices also include offering ideas about what might, or might not, be wrong, either overtly or more tacitly. This practice has been observed in Dutch, U.S., Finnish, Swedish, and British data: for example, patients suggesting a “lay” or “candidate diagnosis” (Houtkoop-Steenstra, 1986), articulating “diagnostic claims” or “etiological hypotheses” (Heritage & Robinson, 2006b), proposing a site of origin for a symptom (Gill & Maynard, 2006), or preemptively resisting a particular diagnosis (Gill et al., 2010). Fragment (3) illustrates this in Swedish data from a study of telephone calls to a nurse-led primary care line where, at Line 3, the caller tentatively suggests a candidate diagnosis in the slot where a request for help might be expected.

**Fragment (3) From Leppänen (2005, p. 184).**

**Figure:**
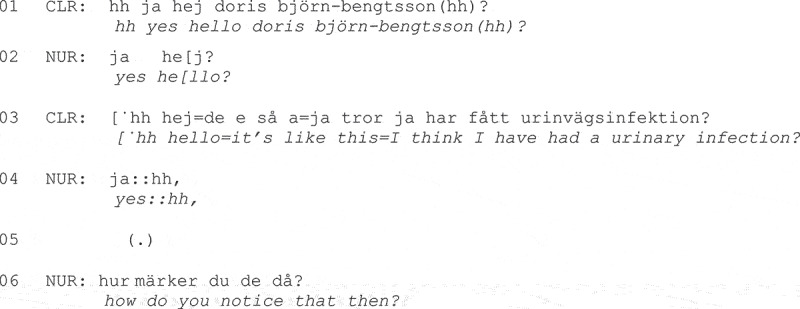

In U.S. data, Gill (1998) reported that patients’ explanations were designed to downplay their knowledge, embedded within speculative questions, or else done in such a way as to avoid setting themselves up for potentially disaffiliative responses. Pomerantz et al. (2007) observed a similar practice in another U.S. dataset in which patients offered medically serious conditions (e.g., a cardiac problem) as a candidate explanation for their symptoms by “displaying some modulated degree of concern or worry” (Pomerantz et al., 2007, p. 144), while at the same time portraying the explanation as unlikely. Exploring patients’ attempts to raise psychological explanations in Danish data, Tarber and Frostholm (2015) found that their success was dependent on physician collaboration. Indeed, a key negative consequence of such a cautious approach is that “physicians may leave patients’ explanations unassessed or even unacknowledged” (Gill & Maynard, 2006, p. 117).

Several studies demonstrated different ways by which primary care staff can be responsive to patients’ ideas about what might be wrong by orienting to candidate diagnoses, either immediately in their next action (as shown by the nurse’s challenge at Line 6 in Fragment (3)), during the physical examination (Heritage & Stivers, 1999), or later on in the diagnostic (Heath, 1992) or treatment phase (Stivers, 2002). Fragment (4), taken from a study of Dutch data, illustrates this below. At the start of the visit (not shown) the patient says, “I think I may have something like a cold,” and informs the physician that he or she had tried to relieve the symptoms with a topical treatment for muscular pain. We are now post-physical examination.

**Fragment (4) From ten Have (1991, p. 8).**

**Figure:**
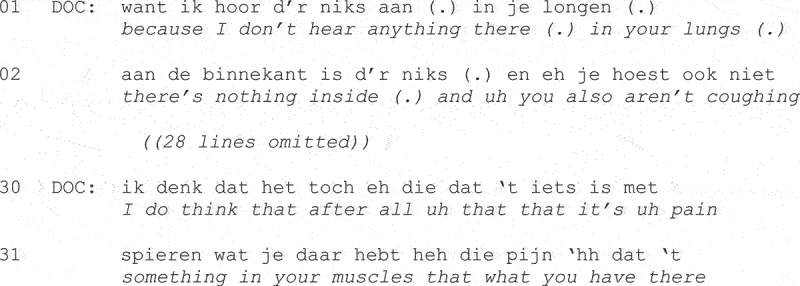

At Line 1 the physician responds to the patient’s earlier idea about what might be wrong, ruling out a respiratory infection with evidence from the physical examination, while later informing the patient at Line 30 that the causal hypothesis implied by the patient’s reported attempt at symptomatic treatment for muscular pain was plausible “after all.”

#### Patients’ concerns

Patients’ agendas can include emotional, social, or psychosocial concerns. Few of our included studies systematically explored patients’ expressions of illness worries or fears in terms of where in the consultation, and how, they were invoked and/or responded to. In Swedish data from nurse-led telephone triage, Leppänen (2005) reported that patients sometimes overtly displayed worry in their problem presentations, taking an “emotional” as opposed to “troubles-resistant” stance. In U.S. data, Stivers and Heritage (2001) observed that patients would sometimes use “narrative expansions” in their responses to physicians’ history-taking questions, allowing for “a volunteering of information that more overtly attends to the patient’s agenda of concerns” (Stivers & Heritage, 2001, p. 167). In Danish data, Tarber (2015) found that patients would often take advantage of moments of “suspended interaction” to disclose “emotional distress induced by social circumstances, bad health or illness worries” (Tarber, 2015, p. 84).

Arreskov et al. (2021) analyzed physician responses to patients’ emotional concerns, or questions expressing such concerns, in Danish data from annual chronic care checkups for patients with multimorbidity. They found that most physicians gave minimal affiliative or non-affiliative responses when patients attempt to introduce a concern, effectively closing them down in favor of progressing the biomedical agenda (Arreskov et al. 2021). The same bias toward progressing a routinized, biomedical “checklist” agenda at the expense of exploring patients’ concerns was observed in British data on nurse-led routine chronic care consultations for diabetes (Rhodes et al., 2006).

#### Patients’ expectations

In many health systems, primary care physicians and nurses play a gate-keeping role, authorizing access to other medical services. Patients therefore often come to a consultation seeking certain treatments or medical interventions they are otherwise unable to access directly (e.g., prescription-only medicines or referrals to specialty care). Several studies identified interactional practices in which a patient/caregiver conveys expectations for particular consultation outcomes. A common finding was that such practices were rarely formatted as direct requests (for instance, Gill, 2005). Instead, they are “frequently interactionally extended and complex, containing a myriad of component actions, such as taking, advocating, and resisting positions regarding the decision, and soliciting and providing information in the service of making the decision” (Robinson, 2001a, p. 20).

In U.S. data from consultations for respiratory illness, Stivers and colleagues have identified a range of more or less overt practices, including “priming,” “nudging,” and “resisting alternative treatments,” by which caregivers of child patients, or adult patients themselves, might be heard (whether intended or not) as advocating for antibiotic treatment (Stivers & Timmermans, 2021). In data from China on pediatric consultations for respiratory illness, overt caregiver advocating actions were more frequently observed than in the U.S. data, with inquiries about antibiotic treatment being the most common (Wang, 2020). Wang (2020) also found that conveying a preference for a particular treatment approach could be used to push back on a physician’s recommendation. This can be seen in Fragment (5). Just prior to this, the caregiver has resisted the physician’s offer of oral antibiotics on the grounds that they have already tried this.

**Fragment (5) Adapted from Wang (2020, p. 10).**

**Figure:**
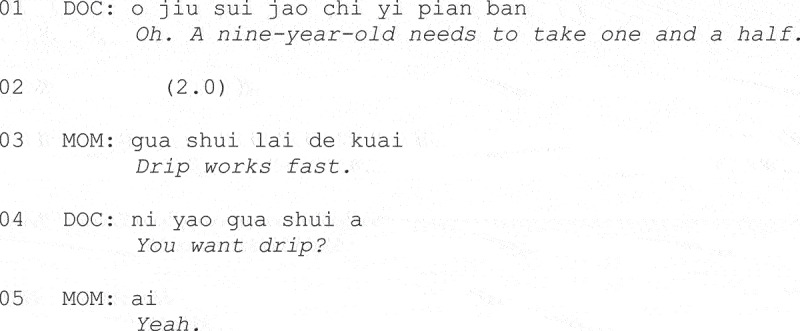

At Line 1 the physician informs the caregiver that the number of tablets her child has been taking was too low. Following two seconds of silence, at Line 3, the patient’s mother conveys her preference for an intravenous antibiotic infusion, thereby educing an offer from the physician at Line 4.

So far, the studies included in our review have shown that, despite being recommended in professional guidance, physicians seldom solicited additional patient problems or concerns. The studies have also shown that patients have multiple ways of voicing their own agendas, ideas, and concerns, although their success in terms of getting them addressed relies wholly on physician collaboration. This finding runs contrary to the wider PHC and professional literature, which recommends that physicians work to discover the “patient’s perspective” (Edwards et al., 2023). Finally, we showed how patients can employ multiple practices to convey expectations for certain consultation outcomes. In the next section we turn to our second theme: managing participation.

## Managing participation

In the wider PHC literature, the concept of participation generally refers to the communicative involvement of the patient during medical visits, particularly in treatment decision making (Robinson, 2003). In this literature, numerous benefits of patient participation have been documented, such that promoting it is now a policy imperative across many Western countries. The implication here being that the default patient position is one of passivity. Our second research theme brings together CA research on how patients and physicians actively manage participation together using language and embodied action as resources. Here, participation is respecified as, “actions demonstrating forms of involvement performed by parties within evolving structures of talk” (Goodwin & Goodwin, 2005, p. 222). This theme brings together a range of interests: (1) studies informed by sociological interests in engagement in collaborative action; (2) studies informed by psychological interests in the revelation and management of affect in terms of how it is embedded in participation; and (3) studies informed by professional/practical interests, such as the impact of technologies or clinical guidelines on participation.

### Engagement in collaborative action

The earliest study in this theme showed how patients can be active agents in collaborative action (Heath, 1982). In British data, Heath observed patients routinely employing embodied “displays of availability” at the start of medical visits, thereby establishing “co-presence” and preparedness to participate (Heath, 1982). Additionally, when physicians were otherwise engaged (e.g., reading the patient’s record), patients were seen to “display recipiency” (i.e., their readiness to reintroduce talk) by gazing toward the physician, thereby eliciting a response (Heath, 1982). Indeed, Heath found that patients employed a range of vocal and embodied actions (e.g., speech perturbation, posture shifts, and gestural activity) either juxtaposed within or alongside an ongoing utterance, to secure physician engagement (Heath, 1984).

Heath also demonstrated how patients used embodied actions in the service of “bodily revelations,” “showing, rather than simply describing” (Heath, 2018, p. 167) the problem, to invite physicians to visually orient to their difficulties. Similarly in Finnish data, Ruusuvuori (2001) demonstrated how patients monitored physician engagement during the problem presentation, treating physician disengagement (e.g., to read or write in the patient record) as problematic. Patients responded with speech “dysfluencies” (e.g., pausing) and/or pointing gestures, in a bid to elicit attention and reestablish mutual involvement (Ruusuvuori 2001, p. 1098). As argued by Heath, “how the doctor attends to and participates in the patient’s talk may be consequential to what the patient says and consequently medical decision-making, treatment programmes and the like” (Heath, 1984, p. 313).

Subsequent U.S. CA studies have evidenced this argument, for example, demonstrating how history-taking sequences (Boyd & Heritage, 2006) can constrain patients’ talk. Yet patients are seldom passive recipients of “designedly restrictive” questions (Stivers & Heritage, 2001). For example, patients’ “expanded answers” during history taking have been shown to preempt possible negative inferences or criticism (Stivers & Heritage, 2001). An example can be seen from British data in Fragment (6), in which the patient, who has presented with “excruciating” hip pain, is asked whether she has taken anything to manage the pain before consulting.

**Fragment (6) From Barnes and Van der Scheer (2021, p. 39).**

**Figure:**
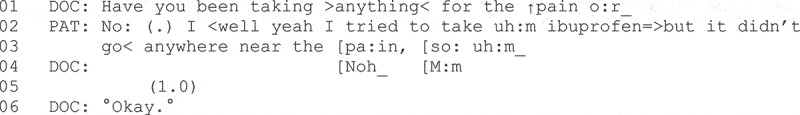

In Line 2, following a turn-initial “no,” the patient’s expanded answer reverses her initial response to “well yeah,” before expanding with a narrative of treatment failure, thereby proclaiming a troubles-resistant stance (Heritage & Robinson, 2006b) and bolstering the legitimacy of their visit.

In other activity phases, patients’ verbal participation has been found to be recurrently patterned. For example, in British data on physical examinations, Heath found that, although cooperative, patients were “seemingly disattentive” to the actions performed, turning away, adopting a “middle-distance orientation,” and withholding any response (Heath, 2006, p. 190). However with pain-related complaints, patients adopted a more active participatory stance to assist in revealing the pain’s location and severity (Heath, 2006). In U.S. data, Heritage and Stivers found that physicians’ “online commentary” practices during the physical examination were “rarely overtly addressed to patients or directly acknowledged by them” (Heritage & Stivers, 1999, p. 1504). Additionally, in a study of the delivery of diagnoses in British data, Heath noted “the absence of patient participation in response” (Heath, 1992, p. 241), unless encouraged, for example, by the physician’s expressions of uncertainty or incongruence with a candidate diagnosis or cause previously implied by patients. In contrast, U.S. data on treatment recommendations have shown they routinely require active patient verbal support and endorsement (Stivers, 2006).

Naturally, there is a need for all parties to negotiate disengagement at the end of the consultation. The role of patient participation in closings has been explored in British and U.S. data—for example, how physical leave taking is coordinated with patients’ acceptance of physicians’ treatment summaries (e.g., “So, the penicillin, one pill four times a day.”) or management plans (e.g., “So, when you come back in a month we’ll check your blood pressure.”) see Heath, 1985; Robinson, 2001b; West, 2006). These “action formulations” (i.e., treatment summaries or management plans) structurally prefer confirmation rather than encouraging any further negotiation (Gafaranga & Britten, 2004, 2007). This would seem, on the face of it, to be appropriate when closing the consultation; however, action formulations are sometimes used without any evidence of prior patient involvement in these plans earlier in the consultation (Gafaranga & Britten, 2004, 2007). Indeed, it is worth noting that “summarizing,” although broadly recommended in the professional literature (Silverman et al., 2013), can have unintended consequences like obstructing further patient talk (Houtkoop-Steenstra, 1986).

Other studies in this theme focused on how verbal participation is managed in “more-than-two-party” consultations—for example, by companions. These studies include PHC interactions with familial caregivers in U.S. pediatric consultations (e.g., Stivers, 2012), and with formal caregivers for patients with intellectual disabilities in British PHC consultations (e.g., Antaki & Chinn, 2019). For reasons of space, we do not discuss these studies here, but for recent reviews that include studies of the role of companions in PHC, see Jenkins et al. (2024) and Webb and Dooley (2024).

### Embedding affect in participation

Several studies in this theme explored how affect is embedded in participation—that is, “the expression of emotions during the consultation, and health professionals’ responses to these displays” (Peräkylä & Ruusuvuori, 2007, p. 173). For example in British data, Heath (2002) showed how patients often employed gestures when describing their symptoms, to express both emotional and personal experiences, such as suffering, that would otherwise remain invisible. The embedding of affect has also been shown to be accomplished more indirectly by patients when orienting to the “delicate” or “sensitive nature” of their talk. For example, Haakana (2001) found in Finnish data that patients used laughter to both display and recognize a problem: “in places where they have to momentarily portray themselves in an unfavorable light” (Haakana, 2001, p. 213). Similarly, Tietbohl and Bergen (2022) showed in U.S. data how patients employed prefaces (e.g., “The thing is … ”) to mark their upcoming talk as disclosing sensitive or embarrassing information when seeking resolution of certain problems. Although not directly displaying emotion as such, patients can be seen as actively managing affect by mitigating the impact of disclosures that might reflect negatively on themselves or the physician.

Regarding responses to displays or expressions of emotion, traditionally, Western physicians have been socialized to adopt a normative stance of “detached concern” (Lief & Fox, 1963), balancing objectivity and empathy. This has been evidenced in British data showing how physicians adopted an objective “analytic stance” in response to patient pain displays in order to fulfill the duties of medical assessment (Heath, 1989). Similarly, in Swedish data, Leppänen (2010) demonstrated how nurses worked to sustain “emotional neutrality” while triaging patients, by accounting for their decisions in organizational and professional terms. However, such a stance can be problematic. To paraphrase Heritage (2011), there can be “dysfunctional” consequences when interactional norms in ordinary conversation (e.g., affiliative responses to troubles talk) are absent during the medical visit. For example, in two separate U.S. studies, Stivers and Heritage (2001) noted that physicians, “may not routinely affiliate with patients’ lifeworld narratives” (Stivers & Heritage, 2001, p. 151), and (Jones, 2001) observed that patients sometimes orient to such absences by leaving a gap or actively pursuing a response.

### The impact of technological innovation on participation

With the rise of technological innovation inside the consulting room, PHC professionals have been increasingly expected to manage “simultaneous and often competing demands” (Heath, 1984, p. 312). A number of early CA studies explored the shift from paper records to electronic health record (EHR) use in terms of how it has shaped and mediated “normal rules of engagement.” For example, in British data, Robinson (1998) demonstrated how embodied shifts in engagement by physicians, in order to accomplish noncollaborative tasks (i.e., the physician reading the EHR), communicated that patient-initiated actions during this time would not be treated as relevant. In data from New Zealand, Dowell et al. (2013) found that transitions into longer episodes of computer use by physicians often featured contemporaneous information produced “online” (e.g., “I’ll just have a look.”). Similarly in Danish data, Beck Nielsen (2014, 2016) noted that physicians often provided “online” explanations to establish the relevance of suspending participation to access supplementary information about patients (e.g., drug allergies), “for the potentially delicate business of validating patients’ own versions” (Beck Nielsen, 2016, p. 72).

In a study of physicians’ prescribing behavior in British data, Greatbatch (2006) revealed a preoccupation with the task-in-hand, in which physicians synchronized the timing of prescription-related talk with related keyboard actions. Moreover, it has been found that patients simultaneously coordinate their turns-at-talk with predictable pauses in ongoing activity in such a way as to minimize possible disruption (Greatbatch, 2006; Greatbatch et al., 1995). Finally, in British data detailing the use of electronic templates in nurse-led routine consultations for chronic disease, Rhodes et al. (2008) found that patient participation was inhibited when nurses did not shift engagement between patient and computer via gaze and bodily orientation.

Other studies have begun to examine the interplay between patients, physicians, and health technologies such as diagnostic instruments and paraclinical tools in PHC consultations (e.g., Ford et al., 2020; Lindell, 2017). For example, Lindell studied how point-of-care testing was introduced, used, and the results accounted for in Danish consultations for common infections (2017). Although designed to assist a physician diagnosis, Lindell found the results—in particular, elevated results—were presented from the position of an unconditional, external authority (2017). These studies of the “communicative use” of health technologies are useful for their critical insights (i.e., actual use vs. planned use and unintended consequences) and the evidence they have provided regarding the impact of technological innovation on participation.

### The impact of professional guidance/policy interventions on participation

As well as illuminating why certain practice guidelines are challenging to implement, CA studies can also reveal unexpected consequences. A small group of studies in this theme focused on the impact of guidelines issued by professional bodies for “good clinical practice” on patient participation. In a British study of “prescribed” questions to assess suicidal ideation, Miller (2013), found that physicians had to work harder to “naturalize” such questions prediagnosis, compared to when they asked them later in the consultation following a diagnosis of depression. They also found that patients had to work hard to mitigate the potentially damaging inferences arising from their responses. As well as the location of these particular questions, their design has also been shown to be interactionally consequential. For example, in another British study, Ford et al. (2021) found that suicide risk assessment questions tended to be “optimized” for a “no-problem” response.

Finally, a small group of studies has considered the successful (or otherwise) diffusion into practice of PHC policy interventions aiming to promote opportunities to support patient self-management. Examples can be seen in a Dutch study of collaborative goal setting and action planning in chronic illness (Lenzen et al., 2018), and a Finnish study on shared decision making (Ijäs-Kallio et al., 2011a). In both studies, the recommended practices were either not observed at all or not implemented as intended by the policy itself. Thereby, a common finding across these studies is a gap between the “theory” and actual practice (see Pilnick, 2022).

In this section we have focused on the management of participation in interaction, bringing together early CA studies of collaborative action with more recent studies exploring the embedding of affect in participation, and studies evaluating the impact of technological innovations and the implementation of talk-based professional/policy interventions. A common feature here has been a multi-modal approach to the analysis of participatory actions. CA studies in this research area have made a unique contribution to the wider primary care literature by demonstrating how the concept of participation can be grounded in actual interaction and providing a robust method for the critical evaluation of prescribed talked-based interventions. In the next section, we explore our final research theme, the management of medical authority in interaction.

## Managing authority

Managing authority has been a longstanding area of interest in medical sociology. The studies included here focused on documenting and understanding authority via the interactional management of “asymmetries” between patients and physicians relating to knowledge (Drew, 1991), initiative (Frankel, 1990), and task (ten Have, 1991). Many of the findings from these studies have clear implications for related professional/practical “problems.” In what follows, we show how CA studies that speak to asymmetries of authority have generated practical insights into four key areas: negotiating shared understanding, giving lifestyle advice, diagnosing illness, and recommending treatment.

### Negotiating shared understanding

Our earliest study in this theme (Meehan, 1981) focused on participant orientations to the unequal distribution of, and rights or entitlement to, medical knowledge. In U.S. data, Meehan explored how the sense of medical terms and procedures could be actively negotiated between physicians and patients. As well as noting cases of self-repair by physicians to address issues of intelligibility when using medical terminology, Meehan illustrated how patients themselves would sometimes initiate repair around physician’s use of specialist vocabulary (e.g., “What is a palpitation?”). Yet, as argued by Drew, “an asymmetry of knowledge is not equivalent to ‘not knowing’” (Drew, 1991, p. 39); many patients regularly use and are familiar with medical terms. Despite this, Meehan (1981) found patients’ own use of medical terms was often mitigated, orienting to their asymmetrical position as a non-authoritative source of that knowledge.

Examining the organization and distribution of “conditionally relevant queries” in U.S. data, West (1984) found these to be evenly distributed between physicians and patients, concluding that they had “mutually accessible means for establishing intelligibility of their utterances” (West, 1984, p. 129). In Fragment (7)—a patient-initiated example—the physician has just checked the patient’s blood pressure.

**Fragment (7) From West (1984, p. 111).**

**Figure:**
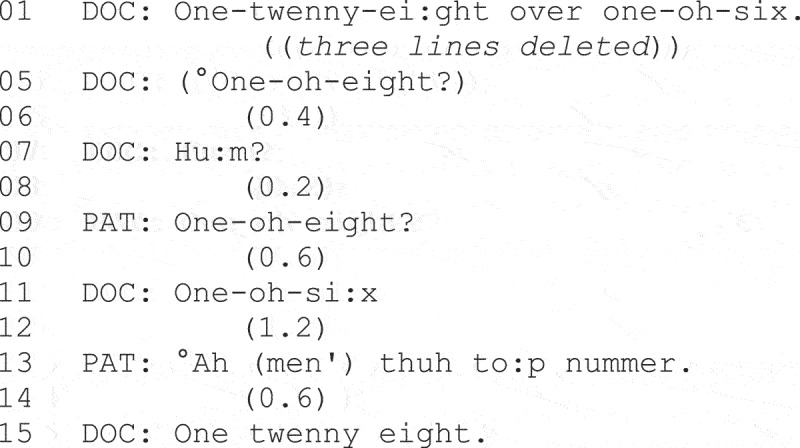

The measurement announced by the physician at Line 1 is subject to a “try-marked” understanding check from the patient, who initiates repair at Line 9. At Line 13, the problem remains unresolved, and the patient locates the repairable in a different way, displaying knowledge that there is a difference between “the top number” and the bottom number.

West also noted that physicians often appended “query terms” (e.g., “Okay?” or “All right?”) to utterances that “offered explanation, gave advice, or proposed plans for future action” (West, 1984, p. 120). Frankel (1990) observed a similar phenomenon, arguing that these physician “solicits” functioned as “last calls,” giving patients the opportunity to confirm or disconfirm intelligibility, or to ask questions before progressing to next activities. However most patient responses consisted of minimal acknowledgment tokens. Another means by which physicians have been shown to attempt to check patient understanding is with “question-seeking questions” (e.g., “Any questions?”). Despite this practice being widely recommended in professional training, Robinson et al. (2016) found in U.S. data that such questions were only asked in 14% (58 out of 407) of visits, and when they were asked, they elicited an affirmative patient response on only 7% of occasions.

In U.S. data on the delivery of test results, Pomerantz and Rintel (2004) demonstrated how patients negotiated unequal distribution of knowledge/expertise by requesting explanations or interpretations of technical medical information when given by physicians without interpretation (e.g., numerical formulations as in Fragment (7)), in order to seek understanding of their import (also see Montenegro & Dori-Hacohen, 2020). Conversely, in a study of blood sugar solicitation sequences in U.S. routine diabetes visits, Wingard et al. (2014) found that physicians would request precise numbers in response to patients’ own non-numerical assessments or interpretations of blood sugar levels (e.g., when indicating no change).

Shared knowledge and understanding between patients and physicians is integral to good medical practice, and its absence may result in the potential for patient harm or litigation. The findings show how the distribution of, and rights or entitlement to, medical knowledge is negotiated in and through interaction between physicians and patients. They also demonstrate *in situ* approaches to monitoring patients’ understanding and their commitment to proposed courses of action.

### Giving lifestyle advice

PHC professionals often need to ask patients about their diet, physical exercise, or alcohol and substance use, in order to establish whether lifestyle may be a factor in their medical problem or to provide advice about ways they can maintain and improve health or wellbeing. This topic is particularly important for patients with chronic illness, as lifestyle changes can have significant impacts on health outcomes. However, because the physician “does not control the knowledge base,” managing epistemic authority in discussions about patients’ personal choices and behaviors can sometimes be “difficult and uncomfortable” (Freeman, 1987, p. 962).

In a study of U.S. data, Freeman found that when physicians made an explicit connection, or personal link, between a patient’s lifestyle and their medical problem, there was less “conversational disruption” (e.g., rejection of the topic). Similar observations have been made in other U.S. data (e.g., Cohen et al., 2011), and in British data (e.g., Connabeer, 2021). In another U.S. study, Bergen (2020) found that when physicians framed advice as “treatment-implicative,” it was more likely to be verbally accepted by patients than “plain advice.” An exception to this pattern was noted in British data, in a study of smoking discussions by Pilnick and Coleman (2003), who found that attempts to personalize a problem that was not shared engendered patient resistance. Relatedly, in Finnish data, Sorjonen et al. (2006) found that physicians were unlikely to give advice to patients regarding their lifestyle choices unless patients had highlighted a problem themselves (and their efforts to change) in their initial response to physician questions about it.

Epistemic rights are linked with personal responsibilities and moral obligations. Several studies examined how these issues were interactionally managed. One common finding was that potential moral implications regarding lifestyle choices or health behaviors can be avoided, or resisted, by patients. Sorjonen et al. (2006) and Denvir (2012) found in Finnish and U.S. data, respectively, that patients would often portray their substance use as “normal and healthy” during history taking, thereby implying its non-problematic character.

**Fragment (8) From Sorjonen et al. (2006, p. 348).**

**Figure:**
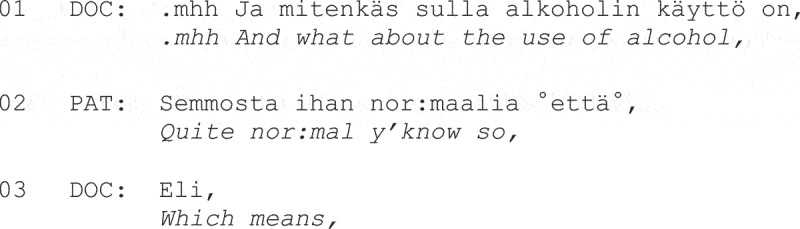

Both studies also found that patients often resisted physicians’ efforts to quantify their substance use with health-oriented descriptions or “no-problem” answers. Sorjonen et al. (2006) found that the latter often resulted in extended sequences due to subsequent physician requests for specification, as shown at Line 3 in Fragment 8.

Possible moral implications were sometimes addressed head on by patients themselves. In Danish data, Guassora et al. (2015) found that, in response to physicians’ questions about lifestyle risks in annual checkups for people with chronic illness, patients would sometimes give anticipatory answers, heading off potential next-turn negative inferences or evaluations by physicians, as shown in Fragment (9).

**Fragment (9) From Guassora et al. (2015, p. 194).**

**Figure:**
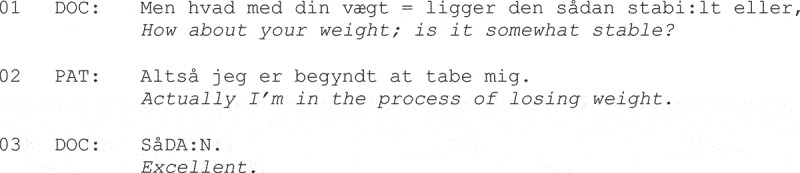

Similarly, in data from New Zealand on diabetes consultations, Barton et al. (2016) found that patients either preemptively blocked likely behavior change advice or resisted it by proffering “candidate obstacles.” These obstacles provided information about the patients’ own life circumstances (e.g., family commitments or other priorities) that might act as a basis for advice resistance or even shape advice given later on in the consultation, while also doing moral work positioning the patient as “willing but unable” (also see Pilnick & Coleman, 2006).

In U.S. data, Dillon (2012) found that patients sometimes engaged in “defensive detailing” as a means of mitigating potential moral implications of disclosed lifestyle behaviors (e.g., in relation to an unplanned pregnancy). Dillon also observed that physicians would sometimes solicit accounts from patients for “medical misdeeds,” “indexing a claim that the accountable event does not accord with common sense and is, thus, possibly inappropriate or unwarranted” (Dillon, 2012, p. 216). However, in U.S. data on chronic care visits, Bergen and Stivers (2013) found that such patient disclosures (e.g., unhealthy behavior choices) could just as commonly be topicalized by patients themselves via “patient-initiated announcements.”

Giving lifestyle advice is widely recognized as challenging for PHC professionals. CA findings in U.S., Nordic, and British data offer clear insights into why this task might be experienced as challenging, and how asymmetries of epistemic authority and “territories of information” are recognized, asserted, and navigated by physicians and patients *in situ*.

### Diagnosing illness

The delivery of a professional opinion in the form of a diagnosis has long been thought to represent the pinnacle of medical authority. In British data recorded in the 1970s and 1980s, Heath found that most diagnostic “informings” were delivered as an authoritative, “factual, monolithic assertion” (Heath, 1992, p. 246) and that, despite opportunities to do so, patients responded minimally (if at all), displaying an “extraordinary passivity” (Heath, 1992, p. 261). More extended patient responses featured if physicians displayed uncertainty or indexed incongruity with the patient’s ideas about what might be wrong, triggering patient’s “post-diagnostic accounts” and expanded diagnostic sequences. For example, bolstering the legitimacy of their grounds for seeking professional help in the face of a “no-problem” diagnosis (Heritage, 2009). Importantly however, patient’s responses “preserved the differential status between their own version and the understanding of the expert” (Heath, 1992, p. 249), underscoring, “the asymmetrical relationship between the participants and the differential status of their opinions concerning illness” (Heath, 1992, p. 264).

In Finnish data collected in the 1990s, Peräkylä (1998) noted a more nuanced balance of authority and accountability. Although “plain assertions” still formed the majority of diagnostic utterances, they were usually delivered immediately adjacent to the physical examination, at which the evidential basis had observably been gathered or could be inferred (Peräkylä, 2006). When delivered at some distance from the physical examination, or when there was diagnostic uncertainty or potential conflict with patients’ own ideas (e.g., regarding the seriousness of their condition), physicians would incorporate more of the evidential basis within what they said (Peräkylä, 2006). Like Heath (1992), Peräkylä (2002) found that extended patient responses (whether agreeing or resisting) only featured if physicians displayed uncertainty, if there was incongruity with the patient’s ideas about what might be wrong, or when the physician explicated the evidential grounds for their diagnosis.

In U.S. data collected between 2003 and 2005, Heritage and McArthur (2019) noted that diagnostic utterances were only present in around 50% of the consultations they examined. Unlike Heath (1992) and Peräkylä (2002), the authors found physicians delivered more “mitigated” diagnostic utterances than plain assertions. However, in line with prior research, they also found that extended patient responses were most likely when diagnoses were epistemically downgraded (Heritage & McArthur, 2019).

In a Finnish study, Ijäs-Kallio et al. (2010) argued that patient’s resistive actions post-diagnosis (such as proffering past experience) indexed a stance that physician epistemic authority was negotiable (also see Drew, 2006). Similarly, in data from China, Wu (2017) found that patients sometimes resisted diagnostic reasoning by drawing on their own authority to contest symptom descriptions. In Danish data, Lindell (2019) found that patients sometimes introduced residual concerns post-diagnosis about symptoms already mentioned but left unexplained or unaddressed (also see Maynard & Frankel, 2019). Although ostensibly the placement of such residual concerns might be seen as resistive, Lindell argued they were more likely a bid for missing relevant information (i.e., concerning symptom treatability, duration, or cause) prior to closing (Maynard & Frankel, 2019).

By providing a detailed picture of the delivery and receipt of diagnostic utterances, CA scholars have enabled a radically different view of the expression and management of medical authority grounded in PHC interactions. Over time and across different healthcare systems, these studies have shown how physicians have moved toward balancing epistemic authority with accountability and uncertainty—moreover, that patients “can, and in a number of cases do, assume a degree of agency and knowledgeability to their diagnosis” (Peräkylä, 2006, p. 246).

### Recommending treatment

Heritage and McArthur (2019) argued that physicians and patients appear to be placing more value/attention on treatment—a solution to the problem presented—rather than diagnosis. As well as embodying epistemic authority, recommending treatment invokes “deontic” authority, the right “to effect changes in the activities of others” (Ervin-Tripp et al., 1984, p. 116). A number of studies have focused on how authority is encoded in treatment directives. In a U.S. study, West first highlighted that treatment directives could be formulated in different ways, ranging from “aggravated” (e.g., orders) to “mitigated” (e.g., suggestions); see West, 1990, p. 86). In Dutch data, ten Have (1995) noted that treatment directives could be formatted via different action types with more or less authority (“proposals,” “announcements,” or “deliberate alternatives”), noting that the choice between the different formats was a likely consequence of the prior sequential environment.

In a comparative study of treatment recommendation-response sequences in U.S. and British PHC data, Stivers et al. (2018) identified five different directive action types (“pronouncements,” “proposals,” “suggestions,” “offers,” and “assertions”). Stivers and colleagues showed how, “with each action type, physicians highlight the recommendation as differentially situated in epistemic and/or deontic space” (Stivers et al., 2018, p. 7), arguing that their selection was shaped by the specific contingencies of the consultation. According to Heritage (2021), the more authoritative treatment recommendations tended to be reserved for medications that were desired by patients, or unproblematic in their administration and side effects, whereas recommendations more oriented to shared decision making were more frequently used for more problematic treatment regimens.

Unlike diagnoses, treatment recommendations are normatively built, and responded to, as a specific next action step to be accepted or rejected, for it is the patient who will ultimately decide whether or not to implement the treatment plan (Stivers, 2005). Several studies have demonstrated how treatment is oriented to as a negotiable matter by both patients and physicians (Stivers, 2006; ten Have, 1995). As ten Have described, during these negotiations “the GP does ‘work’ to put the patient’s acceptance firmly ‘on record,’ as an interactionally-established fact” (ten Have, 1995, p. 324). Yet even in the face of “doctors’ orders,” patients can and do enact agency, “by choosing how and when they endorse the recommendation” (Koenig, 2011, p. 1106). In a study comparing U.S. and British data, Bergen et al. (2018) found contrasting cultural stances toward prescribing by patients and physicians: Whereas American patients were more likely to negotiate for prescription treatment, British patients were more likely to resist, displaying a preference for more cautious prescribing.

In many Western countries, giving patients opportunities to express their views and to play a role in treatment decision making is enshrined in professional guidance. However, in a study of Finnish primary care, Ijäs-Kallio et al. (2011b) noted the “unilateral” character of treatment recommendation sequences, such that patients’ perspectives were seldom elicited. Yet they did find that patients sometimes used expanded responses to voice their own perspectives either in agreement or in negotiating the decision post-recommendation (Ijäs-Kallio et al., 2011b). Koenig has suggested that patient non-acceptance, “represents an opportunity for physicians to explore patient preferences and concerns” (Koenig, 2011, p. 112) post-recommendation. Alternatively, Barnes (2018) has shown how British physicians sometimes do advance work pre-recommendation to explore patient treatment preferences and concerns. Patients’ responses to such preliminary questions often revealed their perspectives on treatment, and physicians were able to subsequently adjust their recommendations, thereby maximizing the chance of acceptance *prima facie* (Barnes, 2018).

Studies in this theme have explored the expression and management of medical authority in PHC consultations. They have provided insights into how physicians and patients collaborate in negotiating medical knowledge and understanding, the challenges inherent in asking about patients’ personal choices and behaviors, that physicians orient to accountability and index uncertainties when delivering diagnoses, and that patients can enact agency in their responses to diagnoses and recommendations for treatment. In what follows, we consider some current and future applications, issues for reflection, and the future of CA research on communication in PHC.

## Applications

The fact that the themes described here all resonate with topics in wider PHC research and professional literature suggests opportunities for clinical, educational, and research applications. CA research has already been at the heart of two PHC interventions in U.S.: a cross-sectional study testing the effectiveness of two experimental questions for upfront agenda-setting in reducing patients’ unmet concerns (Heritage et al., 2007) and a randomized controlled trial testing the effectiveness of a communication-based distance-learning program in reducing inappropriate antibiotic prescribing for respiratory illness (Kronman et al., 2020). There is clearly scope as well for making evidence-based recommendations from CA studies of PHC via “informal” interventions (Robinson & Heritage, 2014, p. 202)—for example, the British “Antimicrobial stewardship out-of-hours” communication training program for primary care professionals (see the OPEN Project, 2021).

Some of the findings from studies reported here (e.g., the design of agenda-setting questions) have already enjoyed exposure in educational settings, featuring in key texts for medical communication skills training (see Silverman et al., 2013). Other findings could usefully inform future clinical communication curricula for both undergraduate medical training and PHC specialty training—for example, specific areas of communication known to be challenging (e.g., discussing sensitive issues or responding to emotion displays), particular clinical topics (e.g., asking about smoking, weight, and alcohol or substance use), giving behavior change advice, and skills in “conducting an effective consultation whilst managing the ‘third party’ presence of the computer” (Noble et al., 2018, p. 1716). The application of CA methods could also play a key role in future implementation research, helping to explain the successes, failures, and unexpected consequences of talk-based policy interventions and providing grounds for new dialogs with policy makers.

## Issues for reflection

PHC entails a continuum from health promotion and disease prevention to treatment, rehabilitation, and palliative care. For practical reasons, we restricted our review to empirical studies of the provision of treatment for minor illnesses and long-term conditions. We also limited our search to studies published in English. Our findings, therefore, should not be seen as representing the field in its entirety. Also, given the number of studies meeting the inclusion criteria, our synthesis was necessarily broad, and some studies were more difficult to place than others. Due to the exploratory nature of our review, it was not appropriate to assess the quality of our included studies. Nevertheless, in collecting and examining the state of knowledge to-date, a number of the field’s strengths and weaknesses have become clear.

In terms of strengths, our review is a testament to the contributions made by the field to mapping the unique interactional “fingerprint” of communication in PHC (Heritage, 2004, p. 225), and providing original insights into topics of longstanding interest to primary care researchers. For example, building on the work of pioneers in the study of ordinary conversation such as Charles Goodwin, CA scholars have provided highly distinctive and compelling characterizations of the embodied collaborative management of patient participation. CA studies of PHC have also helped to expand our methodological reach as a discipline, by demonstrating the power of mixed-methods CA formal coding studies of interaction (e.g., Stivers & Barnes, 2018). Finally, several studies have provided evidence of gaps between professional theories, policies, and “good practice” guidance, and routine practices and actions carried out *in situ*. As argued by Pilnick (2022), these failures can be tracked back to a lack of understanding of how talk works.

Regarding weaknesses in the field, despite the studies originating from 16 different countries/health systems, with data in 17 languages, low- and middle-income countries were hugely under-represented, and four of the languages were English variants. Most data collection was opportunistic and, although provider and patient characteristics may have been collected originally, they were often not adequately reported. When provided, patient demographics usually included age and gender, but less often ethnicity and socioeconomic status. Older patients, children, young people, and persons lacking capacity to consent were under-represented. More precise reporting and embedding inclusion in our study designs would increase the quality and relevance of our work. We also currently rely overwhelmingly on cross-sectional research designs to collect the data on which our observations are based. In order to optimize our contributions, “these observations should ideally be considered in contexts that are both comparative and historical” (Heritage, 2019, p. 111).

## The future of CA research in primary care

Recent years have seen a dramatic increase in remote consulting (Murphy et al., 2021) and changes in the organization and delivery of PHC, due to trends toward task shifting and task sharing with nonmedical professionals (e.g., physician assistants/associates, paramedics, and pharmacists). Building on our cumulative scientific knowledge, future CA research should investigate the impact of these changes. Areas of interest might include the management of participation in telephone consulting (see Seuren et al., 2024) or the management of authority in consultations with nonmedical professionals—for example, how diagnoses and treatment recommendations are delivered, and how they are responded to by patients who may perceive those team members as holding less authority than physicians.

Although our review themes resonate with existing PHC research interests, topics such as the management of patient risk and health inequity were notably absent. Both topics are linked to the quality and experience of care patients receive during contacts with PHC professionals. Future research in these areas should consider employing mixed methods, so that demographic data and post-consultation information about what happened next can be understood in relation to interactional practices and actions. Such an approach would help to identify groups more vulnerable to harm, as well as interactional causes and manifestations of health inequities (see Stivers & Majid, 2007).

Comparative research in different health systems and different cultural contexts can reveal novel insights into global health issues like factors influencing antibiotic prescribing. A collaborative, programmatic approach—as opposed to isolated efforts in individual high-income countries—would enable focused research in distinct workstreams to systematically address different components of an overarching project theme. A good example of this is the, “Touch and affect in health care interaction”^3^^3^See https://projects.tuni.fi/touch/. project, which is analyzing the role of touch and emotion in different types of primary care in three different cultural contexts (Finland, China, and U.K.). We should also consider combining existing datasets or collecting new longitudinal datasets as a window into historical change in primary care, examining how specific interactional practices vary over time and under different sociocultural conditions (Clayman & Heritage, 2021).

## Supplementary Material

Supplemental Material
