# Supplementary material for: Communication in Primary Healthcare: A State-of-the-Art Literature Review of Conversation-Analytic Research
Source: Res Lang Soc Interact. 2024 Apr 3;57(1):7–37. doi: 10.1080/08351813.2024.2305038 (PMC11067862; doi:10.1080/08351813.2024.2305038)
Supplement: Supplemental Material [file HRLS_A_2305038_SM6440.pdf]

	Author/s	Year	Type	Country	HCPs	No.	Patients	No.	Theme<br>1 - Managing agendas<br>2 - Managing participation<br>3 - Managing authority	Data language
1	Heath	1981	Book chapter	UK	Physicians	*	*	*	Managing agendas	British English
2	Meehan	1981	Book chapter	US	Physicians	*	*	*	Managing authority	American English
3	Heath	1982	Article	UK	Physicians	*	*	*	Managing participation	British English
4	Paget	1983	Book chapter	US	Physicians	*	Adults	36	Managing authority	American English
5	West	1983	Book chapter	US	Physicians	18	Mixed	20	Managing authority	American English
6	Heath	1984a	Article	UK	Physicians	*	*	*	Managing participation	British English
7	Heath	1984b	Book chapter	UK	Physicians	*	*	*	Managing participation	British English
8	West	1984	Article	US	Physicians	18	Mixed	20	Managing authority	American English
9	Heath	1985	Article	UK	Physicians	*	*	*	Managing participation	British English
10	Houtkoop-Steenstra	1986	Book chapter	NL	Physicians	1	Adults	1	Managing participation	Dutch
11	Freeman	1987	Article	US	Physicians	*	Adults	200	Managing authority	American English
12	Heath	1989	Article	UK	Physicians	*	*	*	Managing participation	British English
13	Frankel	1990	Book chapter	US	Physicians	*	Adults	10	Managing authority	American English
14	West	1990	Article	US	Physicians	18	Mixed	20	Managing authority	American English
15	ten Have	1991	Book chapter	NL	Physicians	*	*	*	Managing authority	Dutch
16	Heath	1992	Book chapter	UK	Physicians	*	*	*	Managing authority	British English
17	Greatbatch et al.	1993	Article	UK	Physicians	7	*	200	Managing participation	British English
18	Mulholland	1994	Article	AU	Physicians	*	*	*	Managing authority	Australian English
19	Greatbatch et al.	1995	Article	UK	Physicians	7	*	250	Managing participation	British English
20	ten Have	1995	Book chapter	NL	Physicians	*	*	*	Managing authority	British English & Dutch
21	Gill	1998	Article	US	Physicians	4	Adults	15	Managing agendas	American English
22	Peräkylä	1998	Article	FI	Physicians	14	*	100	Managing authority	Finnish
23	Robinson	1998	Article	US	Physicians	9	Mixed	237	Managing participation	American English
24	Heritage & Stivers	1999	Article	US	Physicians	19	Mixed	335	Managing authority	American English
25	Joosten et al.	1999	Article	NL	Physicians	8	Adults	24	Managing participation	Dutch
26	Gill et al.	2001	Article	US	Physicians	1	Adult	1	Managing agendas	American English
27	Haakana	2001	Article	FI	Physicians	*	Adults	60	Managing participation	Finnish
28	Jones	2001	Article	US	Physicians	11	Adults	25	Managing participation	American English
29	Robinson	2001a	Article	US	Physicians	8	*	40	Managing agendas	American English
30	Robinson	2001b	Article	US	Physicians	1	Adult	1	Managing authority	American English

31	Robinson & Stivers	2001	Article	US	Physicians	8	*	40	Managing participation	American English
32	Ruusuvuori	2001	Article	FI	Physicians	10	*	100	Managing participation	Finnish
33	Stivers	2001	Article	US	Physicians	13	Mixed	291	Managing participation	American English
34	Stivers & Heritage	2001	Article	US	Physicians	1	Adult	1	Managing authority	American English
35	Heath	2002	Article	UK	Physicians	*	*	*	Managing authority	British English
36	Manning & Ray	2002	Article	US	Physicians	*	Adults	22	Managing agendas	American English
37	Peräkylä	2002	Article	FI	Physicians	14	Adults	100	Managing authority	Finnish
38	Stivers	2002a	Article	US	Physicians	14	Mixed	360	Managing agendas	American English
39	Stivers	2002b	Article	US	Physicians	14	Mixed	360	Managing agendas	American English
40	Gafaranga & Britten	2003	Article	UK	Physicians	20	Adults	62	Managing agendas	British English
41	Mangione-Smith et al.	2003	Article	US	Physicians	306	Children	10	Managing authority	American English
42	Maynard & Frankel	2003	Book chapter	US	Physicians	1	Adult	1	Managing authority	American English
43	Modaff	2003	Book chapter	US	Physicians	41	Mixed	10	Managing participation	American English
44	Pilnick & Coleman	2003	Article	UK	Physicians	19	Adult	47	3	British English
45	Robinson	2003	Article	US	Physicians	9	Adult	69	Managing participation	American English
46	Stivers et al.	2003	Article	US	Physicians	8	Children	306	Managing agendas	American English
47	Britten et al.	2004	Article	UK	Physicians	20	Mixed	35	Managing participation	British English
48	Campion & Langdon	2004	Article	UK	Physicians	9	*	237	Managing agendas	British English
49	Gafaranga & Britten	2004	Article	UK	Physicians	20	Mixed	62	Managing participation	British English
50	Lutfey	2004	Article	US	Nurses & Physicians	*	Adults	26	Managing authority	American English
51	Pomerantz & Rintel	2004	Article	US	Physicians	*	*	33	Managing authority	American English
52	Collins	2005	Article	UK	Nurses & Physicians	11	Adults	36	Managing authority	British English
53	Gafaranga & Britten	2005	Book chapter	UK	Physicians	20	Mixed	62	Managing agendas	British English
54	Gill	2005	Article	US	Physicians	1	Adult	1	Managing agendas	American English
55	Kitzinger	2005	Article	UK	Physicians	1	*	59	not highlighted	British English
56	Leppänen	2005	Book chapter	SE	Nurses	13	Mixed	276	Managing agendas	Swedish
57	Robinson & Heritage	2005	Article	US	Physicians	77	*	302	Managing agendas	American English
58	Stivers	2005a	Article	US	Physicians	38	Children	540	Managing authority	American English
59	Stivers	2005b	Article	US	Physicians	14	Children	360	Managing authority	American English
60	Wynn	2005	Article	NO	Physicians	3	Adults	77	Managing participation	British English
61	Boyd & Heritage	2006	Book chapter	US	Physicians	1	Adult	1	Managing agendas	American English

62	Drew	2006	Book chapter	UK	Physicians	1	*	60	Managing agendas	British English
63	Gill & Maynard	2006	Book chapter	US	Physicians	5	Adults	15	Managing agendas	American English
64	Greatbatch	2006	Book chapter	UK	Physicians	4	*	80	Managing participation	British English
65	Halkowski	2006	Book chapter	US	Physicians	*	Adults	25	Managing agendas	American English
66	Heath	2006	Book chapter	UK	Physicians	*	*	*	Managing participation	British English
67	Heritage & Robinson	2006a	Article	US	Physicians	77	*	302	Managing agendas	American English
68	Heritage & Robinson	2006b	Book chapter	US	Physicians	*	*	300	Managing agendas	American English
69	Mangione-Smith et al.	2006	Article	US	Physicians	38	Children	540	Managing authority	American English
70	Maynard & Frankel	2006	Book chapter	US	Physicians	*	*	*	Managing authority	American English
71	Peräkylä	2006	Book chapter	FI	Physicians	14	*	100	Managing authority	Finnish
72	Pillet-Shore	2006	Article	US	Nurses	*	Adults	14	Managing authority	American English
73	Pilnick & Coleman	2006	Article	UK	Physicians	19	Adults	47	Managing authority	British English
74	Rhodes et al.	2006	Article	UK	Nurses	1	Adult	1	Managing participation	British English
75	Robinson & Heritage	2006a	Article	US	Physicians	28	Adults	182	Managing agendas	American English
76	Robinson & Heritage	2006b	Book chapter	US	Physicians	28	Adults	142	Managing agendas	American English
77	Sorjonen et al.	2006	Book chapter	FI	Physicians	14	Adults	90	Managing authority	Finnish
78	Stivers	2006	Book chapter	US	Physicians	*	*	*	Managing authority	American English
79	West	2006	Book chapter	US	Physicians	15	Mixed	62	Managing agendas	American English
80	Cahill & Papageorgiou	2007	Article	UK	Physicians	16	Children	32	Managing participation	British English
81	Gafaranga & Britten	2007	Book chapter	UK	Physicians	62	Mixed	20	Managing participation	British English
82	Pomerantz et al.	2007	Book chapter	US	Physicians	3	Adults	3	Managing agendas	American English
83	Stivers & Majid	2007	Article	US	Physicians	38	Children	570	Managing participation	American English
84	Dew et al.	2008	Article	NZ	Physicians	9	Adults	105	Managing authority	New Zealand English
85	Rhodes et al.	2008	Article	UK	Nurses & Physicians	13	Adults	26	Managing participation	British English
86	Ariss	2009	Article	UK	Physicians	4	Adults	23	Managing agendas	British English
87	Frers	2009	Article	DE	Physicians	*	*	*	Managing participation	American English
88	Cahill	2010	Book chapter	UK	Physicians	16	Children	32	Managing participation	British English
89	Gill et al.	2010	Article	US	Physicians	*	Adults	50	Managing agendas	American English
90	Heritage et al.	2010	Article	US	Physicians	38	Children	570	Managing authority	American English
91	Hewitt et al.	2010	Article	UK	Physicians	18	Adults	65	Managing participation	British English
92	Ijäs-Kallio et al.	2010a	Article	FI	Physicians	11	Mixed	10	Managing authority	Finnish

93	Ijäs-Kallio et al.	2010b	Article	FI	Physicians	11	Mixed	86	Managing agendas	Finnish
94	Leppänen	2010	Book chapter	SE	Nurses	13	Mixed	276	Managing participation	Swedish
95	Newman et al.	2010	Article	UK	Physicians	4	*	52	Managing participation	British English
96	Pilnick & Coleman	2010	Article	UK	Physicians	19	Adults	26	Managing authority	British English
97	Cohen et al.	2011	Article	US	Physicians	28	Adults	541	Managing authority	American English
98	Ijäs-Kallio et al.	2011	Article	FI	Physicians	11	Mixed	86	Managing participation	Finnish
99	Koenig	2011	Article	US	Physicians	*	Mixed	100	Managing authority	American English
100	Beck Nielsen	2011	Article	DK	Physicians	4	Adults	52	Managing agendas	Danish
101	Park	2011	Article	KR	Physicians	6	*	42	Managing participation	Korean
102	Denvir	2012	Article	US	Physicians	6	Adults	24	Managing authority	American English
103	Dillon	2012	Article	US	Physicians	15	Adults	96	Managing authority	American English
104	Beck Nielsen	2012	Article	DK	Physicians	4	Adults	52	Managing agendas	American English
105	Stivers	2012	Article	US	Physicians	38	Children	570	Managing participation	American English
106	Bergen & Stivers	2013	Article	US	Physicians	*	*	57	Managing agendas	American English
107	Dowell et al.	2013	Article	NZ	Physicians	10	Adults	28	Managing participation	New Zealand English
108	Halkowski & Beach	2013	Book chapter	US	Physicians	*	Adults	100	Managing authority	American English
109	Miller	2013	Article	UK	*	*	*	*	Managing participation	British English
110	Park	2013	Article	KR	Physicians	7	Adults	60	Managing agendas	Korean
111	Chatwin et al.	2014	Article	UK	Nurses & Physicians	14	Adults	26	Managing agendas	British English
112	Beck Nielsen	2014	Book chapter	DK	Physicians	4	*	52	Managing participation	Danish
113	Wingard et al.	2014	Book chapter	US	Physicians	*	*	55	Managing authority	American English
114	Guassora et al.	2015	Article	DK	Physicians	6	Adults	15	Managing authority	Danish
115	Guzmán	2015	Article	CL	Healers & Physicians	5	Adults	45	Managing authority	Mapudungun & Spanish
116	Mangione-Smith et al.	2015	Article	US	Physicians	28	Children	100	Managing authority	American English
117	Tarber	2015	Article	DK	Physicians	10	Adults	3	Managing agendas	Danish
118	Tarber & Frostholm	2015	Article	DK	Physicians	10	Adults	187	Managing agendas	Danish
119	Wheat et al.	2015	Article	UK	Physicians	13	Adults	49	Managing authority	British English
120	Barton et al.	2016	Article	NZ	Allied HCPs, Nurses & Physicians	*	Adults	34	Managing authority	New Zealand English
121	Beck Nielsen	2016	Article	DK	Physicians	4	*	52	Managing participation	Danish

122	Robinson et al.	2016	Article	US	Physicians	85	Adults	407	Managing agendas	American English
123	Tarber & Frostholm	2016	Article	DK	Physicians	10	Adults	187	Managing authority	Danish
124	Vickers et al.	2016	Article	US	Nurses & Physicians	5	Adults	50	Managing participation	American English & Spanish
125	Lindell	2017	Article	DK	Physicians	15	Mixed	86	Managing participation	Danish
126	Park	2017	Article	KR	Physicians	7	Adults	24	Managing agendas	Korean
127	Wu	2017	Article	CN	Physicians	6	Mixed	100	Managing authority	Mandarin
128	Barnes	2018	Article	UK	Physicians	36	Adults	57	Managing participation	British English
129	Bergen et al.	2018	Article	US	Physicians	93	Mixed	697	Managing authority	American & British English
130	Heath	2018	Book chapter	UK	Physicians	*	*	*	Managing participation	British English
131	Lenzen et al.	2018	Article	NL	Nurses	3	Adults	5	Managing participation	Danish
132	McArthur	2018	Article	US	Physicians	10	Adults	255	Managing participation	American English
133	Beck Nielsen	2018a	Article	DK	Physicians	4	Adults	52	Managing participation	Danish
134	Beck Nielsen	2018b	Article	DK	Physicians	4	Adults	52	Managing authority	Danish
135	Beck Nielsen	2018c	Article	DK	Physicians	4	Adults	52	Managing participation	Danish
136	Nguyen & Austin	2018a	Article	AU	Physicians	12	Adults	31	Managing authority	Vietnamese
137	Nguyen & Austin	2018b	Article	AU	Physicians	15	Adults	66	Managing agendas	Vietnamese
138	Stivers et al.	2018	Article	US	Physicians	*	*	*	Managing authority	American English
139	Abu-Elrob	2019	Article	UK	Physicians	8	Mixed	20	Managing participation	Arabic
140	Antaki & Chinn	2019	Article	UK	Nurses & Physicians	14	Adults	33	Managing participation	British English
141	Cabral et al.	2019	Article	UK	Nurses & Physicians	13	Children	60	Managing agendas	British English
142	Cheng	2019	Article	HK	Nurses	5	Adults	61	Managing participation	Cantonese & Mandarin Chinese
143	Chinn	2019	Article	UK	Nurses & Physicians	14	Adults	33	Managing participation	British English
144	Heritage & McArthur	2019	Journal article	US	Physicians	71	Mixed	255	Managing authority	American English
145	Lindell et al.	2019	Book chapter	DK	Physicians	15	Mixed	86	Managing agendas	Danish
146	McArthur	2019	Article	US	Physicians	10	Adults	255	Managing authority	American English
147	Beck Nielsen	2019a	Article	DK	Physicians	4	Adults	52	Managing participation	Danish
148	Beck Nielsen	2019b	Book chapter	DK	Physicians	1	Adults	1	Managing agendas	Danish

149	Stevenson et al.	2019	Article	UK	Physicians	5	Mixed	18	Managing participation	British English
150	Tate	2019	Article	US	*	*	Adults	14	Managing agendas	American English
151	Wang	2019	Book chapter	CN	Physicians	9	*	196	Managing participation	Mandarin Chinese
152	Abu-Elrob	2020	Article	UK	Physicians	8	Mixed	20	Managing participation	Arabic
153	Bergen	2020	Article	US	Physicians	12	Adults	48	Managing authority	American English
154	Ford et al.	2020	Article	UK	Physicians	*	Adults	2	Managing participation	British English
155	Kushida et al.	2020	Article	JP	Physicians	19	Adults	51	Managing agendas	Japanese
156	La et al.	2020	Book chapter	NZ	Physicians	*	Adults	24	Managing participation	New Zealand English
157	Li	2020	Article	CN	Physicians	*	*	8	Managing authority	Mandarin Chinese
158	Montenegro & Dori-Hacohen	2020	Article	US	Physicians	9	Adults	10	Managing authority	American English
159	Wang	2020	Article	CN	Physicians	9	*	196	Managing agendas	Mandarin Chinese
160	Arreskov et al.	2021	Article	DK	Physicians	13	Adults	15	Managing agendas	Danish
161	Barnes & van der Scheer	2021	Book chapter	UK	Physicians	23	Adults	134	Managing authority	British English
162	Chinn & Ruddall	2021	Article	UK	Nurses & Physicians	14	Adults	33	Managing participation	British English
163	Connabeer	2021	Article	UK	Physicians	23	Adults	85	Managing authority	British English
164	Erkelens et al.	2021	Article	NL	Nurses	*	Adults	68	Managing participation	Dutch
165	Ford et al.	2021	Article	UK	Physicians	*	Adults	52	Managing participation	British English
166	McCabe	2021	Article	UK	Physicians	*	Adults	23	Managing authority	British English
167	Ostermann	2021	Article	BR	Physicians	3	*	103	Managing authority	Brazilian Portuguese
168	Stevenson et al.	2021a	Article	UK	Physicians	10	Adults	28	Managing authority	British English
169	Stevenson et al.	2021b	Article	UK	Physicians	10	Adults	28	Managing authority	British English
170	Stivers & Timmermans	2021	Article	US	Physicians	42	*	68	Managing agendas	American English
171	Stortenbeker et al.	2021	Article	NL	Physicians	9	Adults	36	Managing participation	Dutch
172	Wang & Liu	2021	Article	CN	Physicians	9	*	196	Managing authority	Mandarin Chinese
173	Weatherall et al.	2021	Article	NZ	Physicians	6	Adults	9	Managing participation	New Zealand English
174	Chinn	2022	Article	UK	Nurses & Physicians	14	Adults	33	Managing participation	British English
175	Dooley & Barnes	2022	Article	UK	Paramedics & Physicians	4	Adults	17	Managing agendas	British English
176	Tietbohl	2022	Article	US	Physicians	4	Adults	52	Managing participation	American English

177	Tietbohl & Bergen	2022	Article	US	Physicians	14	Adults	90	Managing participation	American English

Country of origin abbreviations:

AU – Australia
BR – Brazil
CL – Chile
CN – China
DE – Germany
DK – Denmark
FI – Finland
HK – Hong Kong
JP – Japanese
KR – Korea
NL – Netherlands
NO – Norway
NZ – New Zealand
SE – Sweden
UK – United Kingdom
US – United States
